# A Commercial Anti-TIF1γ ELISA Is Superior to Line and Dot Blot and Should Be Considered as Part of Routine Myositis-Specific Antibody Testing

**DOI:** 10.3389/fimmu.2022.804037

**Published:** 2022-01-28

**Authors:** Ben Mulhearn, Danyang Li, Fionnuala McMorrow, Hui Lu, Neil J. McHugh, Sarah L. Tansley

**Affiliations:** ^1^Department and Pharmacy and Pharmacology, University of Bath, Bath, United Kingdom; ^2^Royal National Hospital for Rheumatic Diseases, Royal United Hospitals, Bath, United Kingdom

**Keywords:** TIF1γ, cancer, autoantibodies, myositis, ELISA - enzyme-linked immunosorbent assay, myositis - diagnosis, dermatomysitis

## Abstract

**Objectives:**

Anti-TIF1γ is an important autoantibody in the diagnosis of cancer-associated dermatomyositis and the most common autoantibody in juvenile onset dermatomyositis. Its reliable detection is important to instigate further investigations into underlying malignancy in adults. We previously showed that commercial assays using line and dot blots do not reliably detect anti-TIF1γ. We aimed to test a new commercial ELISA and compare with previously obtained protein immunoprecipitation.

**Methods:**

Radio-labelled immunoprecipitation had previously been used to determine the autoantibody status of patients with immune-mediated inflammatory myopathies and several healthy controls. ELISA was undertaken on healthy control and anti-TIF1γ sera and compared to previous immunoprecipitation data.

**Results:**

A total of 110 serum samples were analysed: 42 myositis patients with anti- TIF1γ and 68 autoantibody negative healthy control sera. Anti-TIF1γ was detected by ELISA in 41 out of 42 of the anti-TIF1γ-positive samples by immunoprecipitation, and in none of the healthy controls, giving a sensitivity of 97.6% and specificity of 100%. The false negative rate was 2%.

**Conclusion:**

ELISA is an affordable and time-efficient method which is accurate in detecting anti-TIF1γ.

## Highlights

1. Anti-TIF1γ is a key autoantibody in the diagnosis of cancer-associated dermatomyositis and juvenile dermatomyositis
2. ELISA is a quick and easy method in accurately detecting anti-TIF1γ autoantibodies
3. Diagnosis of IIMs should include ANA immunofluorescence, line or dot blot, and anti-TIF1γ ELISA

## Introduction

The ability to detect myositis -specific and -associated antibodies (MSAs and MAAs), which can be found in the sera of 60 – 70% patients with immune-mediated inflammatory myopathies (IIMs) (1), has greatly improved the diagnosis and phenotyping of these rare diseases. Not only do they aid diagnosis, but they also guide further investigation and management (2). For instance, it is well-known that IIMs, and dermatomyositis (DM) in particular, are strongly linked with cancer, with estimates varying between 7 and 32% (3).

Anti-transcription intermediary factor 1γ (TIF1γ) autoantibodies are found in both juvenile dermatomyositis (JDM) and adult IIMs. They are present in 7% of European adults with DM and 20 – 30% of children affected by JDM (2). Strikingly, 38 – 84% of patients adult DM patients ≥ 39 years of age who are TIF1γ-positive in both European and Japanese cohorts develop cancer in the 3 years before and after DM diagnosis (4–6). Anti-TIF1γ detection in patients with a new diagnosis of DM ≥ 39 years of age may therefore prompt a thorough investigation for the detection of cancer and reduce cancer mortality rates, making the accurate detection of anti-TIF1γ a research priority.

Currently the reference standard in the detection of MSAAs is immunoprecipitation (IP) due to its ability to detect well-described and novel autoantibodies. However, this technique is impractical for use in clinical practice owing to its expense and the length of time it takes to reach a result which usually takes a minimum of 2 – 3 weeks. For this reason, several commercially available immunoassays have become available which are low cost, easy to use, and are reported to detect an array of MSAAs. However, these immunoassays are subject to both false positives and false negatives. A number of them have recently been tested by our group and others (7, 8). In particular, anti-TIF1γ was found to be particularly problematic with false negatives found in 40% samples analysed by line blot and 76% by dot blot (7). Espinosa-Ortega et al. (8) also found low concordance between anti-TIF1γ detected by line/dot blot and immunoprecipitation, with a Cohen’s kappa of 0.56. This is likely because anti-TIF1γ frequently target a conformational epitope, meaning the tertiary antigen structure is required to remain intact to be recognised by the autoantibody (9). Whereas line and dot blot immunoassays utilise denatured antigen, enzyme-linked immunosorbent assays (ELISAs) maintain the tertiary structure of the protein. Fujimoto et al. (10) recently tested a newly-developed ELISA in a Japanese cohort of patients with a spectrum of IIMs, and found this approach to be highly effective with 100% sensitivity and specificity which was a result comparable to immunoprecipitation.

In this study, we aimed to test the same commercial ELISA kit (Medical & Biological Laboratories Co. Ltd., Nagoya, Aichi, Japan) for the detection of TIF1γ autoantibodies in a European cohort of adult IIM patients and compared results with samples previously analysed using immunoprecipitation.

## Methods

### Sample Selection

Myositis serum samples used in this study were chosen as previously described (7) from a biobank of more than 3000 samples collected for research or diagnostic purposes (2, 11). All serum samples had previously been analysed by immunoprecipitation locally and contain at least one MSAA. Twenty-five anti-TIF1γ samples had also been previously analysed by line and dot blot (7). Briefly, sera were stored at -20°C prior to analysis in a facility at the University of Bath. The study had ethical approval through the host Institute (University of Bath EIRA reference number 17-01211). All samples from research cohorts had existing ethics in place.

### ELISA

ELISA was performed on 5µL of diluted serum sample as per the manufacturer’s instructions (Medical & Biological Laboratories Co. Ltd., Nagoya, Aichi, Japan). All samples were run in duplicate. Briefly, samples were thawed and diluted to a 1:101 concentration and incubated on a microwell plate for 30 minutes. Wells were then incubated with a horseradish peroxidase-conjugated goat anti-human IgG antibody conjugate for 30 minutes followed by a TMB/peroxide substrate for 15 minutes. The reaction was terminated by 0.25 mol/L sulfuric acid. All incubations took place at room temperature with 4 wash cycles between steps. The absorbance of each well was read on a FLUOstar Omega microplate reader (BMG Labtech Ltd., Aylesbury, Buckinghamshire, Great Britain) at 450 nm wavelength. Positive and negative cut off values were calculated according to previous work described by Fujimoto et al. (10) and expressed in arbitrary units (au).

### Immunoprecipitation

Radio-immunoprecipitation had been previously undertaken as described by Tansley et al. (7). Briefly, sera were mixed with protein-A-Sepharose beads and a 35(S)methionine radiolabelled K562 cell extract, followed by fractionation by SDS-PAGE and analysis by autoradiography. A characteristic doublet band at 155/140 was read as being positive for TIF1γ (12).

### Data Analysis

Statistical analysis was undertaken using Prism 9 version 9.2.0 for macOS (GraphPad Software, LLC., San Diego, CA, USA). Confidence intervals (CI) are expressed at 95%.

## Results

A total of 110 serum samples were analysed, of which 42 were known to have anti-TIF1γ and 68 were healthy control sera. Immunoprecipitation data was held for all samples. Diagnoses included DM (n=27), clinically amyopathic DM (n=4), JDM (n=5), polymyositis (n=4), and overlap syndrome (n=2). All HC samples tested were autoantibody negative by immunoprecipitation.

### Commercial TIF1γ ELISA Performed as Well as Immunoprecipitation

Forty-one patient samples with anti-TIF1γ tested positive by ELISA as defined by a cut-off point of 32 au. None of the HC samples tested positive using this cut-off point. The remaining anti-TIF1γ positive sample was just under the cut-off for positivity (30.2 au). This gives an area under the ROC curve (AUC) of 0.988 (CI 0.961 – 1.000, P < 0.0001) which is equivalent to sensitivity of 97.6% (CI 87.7% - 99.9%) and a specificity of 100% (CI 94.65% - 100%). In this case, Cohen’s Kappa would give a value of 1.

**Figure 1:**
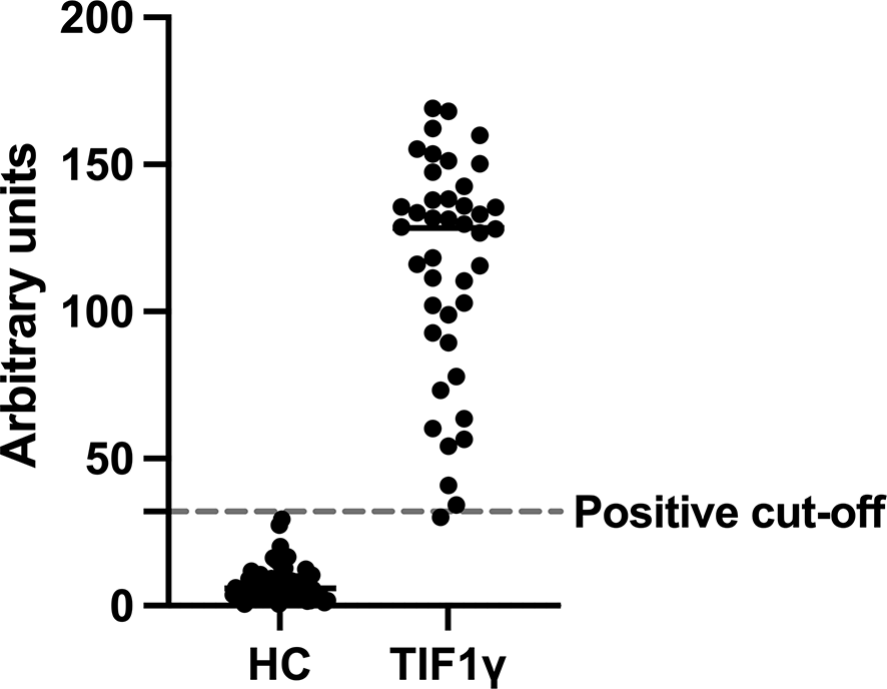
TIF1γ ELISA values for 68 healthy controls and 42 TIF1γ serum samples.

Quantitative results for the ELISA values are shown in **Figure 1**. Briefly, the median ELISA assay result for HC samples was 5.99 au. (median CI 4.74 – 7.87) and for the TIF1γ samples was 128.5 au. (median CI 110.4 – 135.4).

Graph showing the relative ELISA titres for healthy control and TIF1γ samples expressed in arbitrary units for each individual serum sample (circles). Dashed line represents the positive cut-off point as previous described (10). All 68 healthy control (HC) samples were underneath the cut-off and all but one of the 42 TIF1γ samples were above the cut-off. The TIF1γ sample below the cut-off had a weak band in the 140/155 kDa region.

### Low Anti-TIF1γ ELISA Titres Are Associated With False Negative Line Blot Results

**Figure 2:**
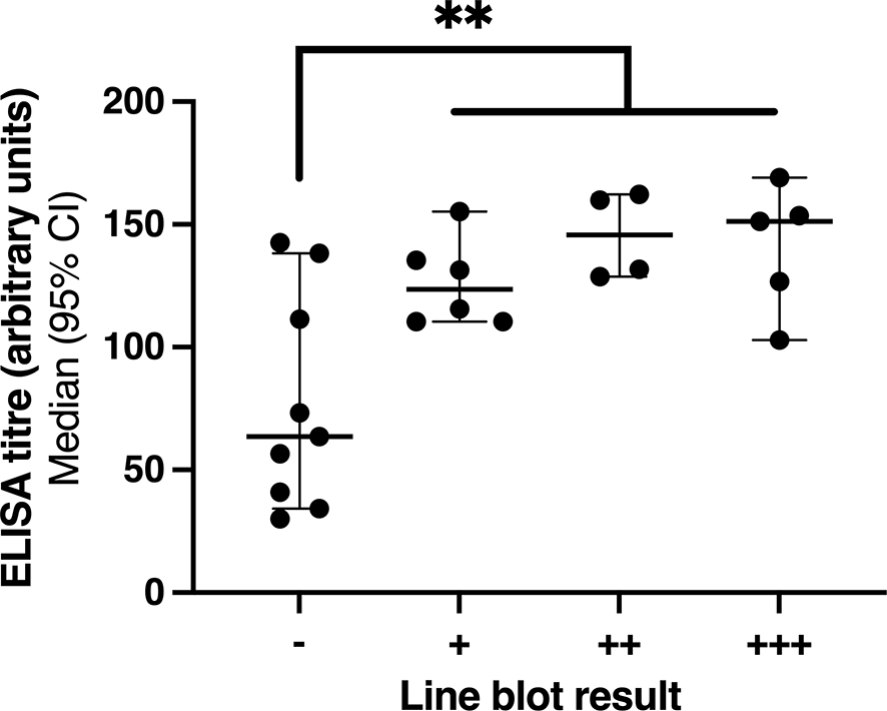
Comparison of anti-TIF1γ ELISA titre and line blot result. **P ≤ 0.01.

Given that our group previously tested 25 anti-TIF1γ samples by line blot, we were able to compare ELISA titres in this study with this data to try and understand which samples might test negative by line blot. The results are shown in **Figure 2**. All anti-TIF1γ positive samples by ELISA with low titres (between 30 – 100 au.) tested negative by line blot. However, 3 out of the 9 samples testing negative by line blot had high anti- TIF1γ titres (> 100 au.). The difference in ELISA titres between those testing negative and positive by line blot was statistically significant (P = 0.0041, two-tailed Mann-Whitney test), suggesting that lower anti-TIF1γ antibody titres lead to false negative line blot results. Similarly, dot blot samples returned only 7/24 (29%) true positives out of the anti-TIF1γ samples that tested positive by ELISA and immunoprecipitation.

Graph showing a comparison between anti-TIF1γ ELISA titre and line blot result, as previously tested by our group (7). ELISA titres are expressed in arbitrary units and calculated as per the manufacturer’s instructions. Lines and error bars represent median values with 95% confidence intervals. The line blot results are expressed as negative (–), low positive (+), moderately positive (++), and high positive (+++). The median ELISA values for negative, low positive, moderately positive, and high positive results were 63.5 au., 123.5 au., 145.8 au., and 151.2 au., respectively. A two-tailed Mann Whitney test comparing ELISA titres between negative (–) and positive (+, ++, +++) line blots found a statistical difference between the two groups (P = 0.0041).

## Discussion

This data has shown that accurate detection of anti-TIF1γ can be achieved by ELISA and confirms the findings made by Fujimoto et al. (10). The accuracy of detection is high and would be acceptable for use in clinical practice. Compared to other cost- and time- effective methods such as line and dot blot which have false negative rate of 40% - 70% (7), this data found that ELISA has a false negative rate of 1/42 (2%). This data has also shown that anti-TIF1γ titre correlates with a positive line blot result. This result is not unexpected given that the line blot is a semi-quantitative method of detecting autoantibodies. Importantly, where ELISA was able to detect samples with low titres of anti-TIF1γ (between 30 – 100 au.), line blot was unable to do so. Line blot also failed to detect some samples with high anti-TIF1g titres (> 100 au.). Taken together, anti-TIF1γ ELISA performs better than line blot in detecting this clinically important autoantibody.

Anti-TIF1γ status by immunoprecipitation was determined by recognition of 155/140kDa bands alongside an anti-TIF1γ standard control. It remains possible that the sample negative by ELISA has an unknown autoantibody with an identical band pattern although this would seem unlikely. Furthermore, the sample produced an ELISA result just below the positive threshold and may simply be a low-titre positive. The ELISA threshold could be adjusted to reduce the likelihood of this occurring, but this is likely to lead to some false positives. The most appropriate cut-off threshold may depend on the clinical context, for example, a low false positive rate may be tolerable in patients with confirmed dermatomyositis to inform the intensity of malignancy screening.

The current study was not designed to investigate the relationship between anti-TIF1γ titres and cancer detection rates. It would, however, be useful to investigate how anti-TIF1γ titre using ELISA correlates with malignancy. Recent work by Fiorentino et al. (13) found anti-TIF1γ titre positively correlated with cancer detection rate in DM, ranging from 8% detection for low titres to 36% detection for high titres. Furthermore, some of our healthy control samples had low anti-TIF1γ titres just below the positive cut-off and it would be of interest to investigate if these healthy subjects had a higher malignancy rate compared to a negative anti-TIF1γ control population.

The detection of anti-TIF1γ in adult DM patients should be considered a red flag for malignancy (4–6). Accurate and timely detection of anti-TIF1γ autoantibodies is therefore vital for these patients to ensure underlying malignancy is diagnosed and treated promptly. We suggest that, when investigating IIMs, anti-TIF1γ ELISA is undertaken alongside, ANA testing and a multiplex immunoblot assay to ensure accurate detection of this important autoantibody.

## Data Availability Statement

The raw data supporting the conclusions of this article will be made available by the authors, without undue reservation.

## Ethics Statement

The studies involving human participants were reviewed and approved by University of Bath EIRA 17-01211. Written informed consent to participate in this study was provided by the participants’ legal guardian/next of kin.

## Author Contributions

Experiments were carried out by BM, HL, FM, and DL. Manuscript was drafted by BM and read by ST and NM. All authors contributed to the article and approved the submitted version.

## Funding

This work was supported by funding from CureJM and the Bath Institute of Rheumatic Diseases. ELISA kits were provided by MBL.

## Conflict of Interest

The authors declare that the research was conducted in the absence of any commercial or financial relationships that could be construed as a potential conflict of interest.

## Publisher’s Note

All claims expressed in this article are solely those of the authors and do not necessarily represent those of their affiliated organizations, or those of the publisher, the editors and the reviewers. Any product that may be evaluated in this article, or claim that may be made by its manufacturer, is not guaranteed or endorsed by the publisher.
